# State Control of Hog-cholera1Read before the Thirty-fifth Annual Meeting of the United States Veterinary Medical Association, September, 1898.

**Published:** 1898-09

**Authors:** M. H. Reynolds

**Affiliations:** St. Anthony Park, Minn.


### STATE CONTROL OF HOG-CHOLERA.[1]

By M. H. Reynolds, D.V.M.,
ST. ANTHONY PARK, MINN.

It has been a belief rather generally accepted among State authorities working with infectious diseases of animals that hog-cholera was just hog-cholera, a sort of hopeless job anyhow, and that there was no use of attempting anything in the way of State control. I wish to insist that such lethargy is not justifiable, and that State authorities that are not making an organized and aggressive fight against hog-cholera are not doing their full duty. It is a common excuse that "we have no such laws back of us as you have in Minnesota." The simple remedy of this difficulty is to

[1] Read before the Thirty-fifth Annual Meeting of the United States Veterinary Medical Association, September, 1898.

get such laws. I am fully aware that the hog-cholera problem is a large one. I am also aware from personal experience in a long struggle that it is a difficult one; but I still insist that under favorable conditions it is both possible and practical to quarantine hog-cholera.

My discussion of this problem must necessarily be largely of Minnesota methods, for, so far as I know, Minnesota is the first State that has made any definite, organized attempt to control extensive outbreaks of this disease.

We have a State Board of Health, consisting of eight physicians and one veterinarian, as a central body, and 1841 local health officers and local boards of health. (Boards of township supervisors are *ex-officio* local boards of health in the country.) Our State Board of Health, so called, together with every local health officer in the State, constitutes the real State Board of Health. This makes an immense but fairly well-organized machine.

Our work in the State Board of Health is divided into three departments: secretary and general executive officer; bacteriological laboratory and director, and veterinary department and director. The fund appropriated for the work with infectious diseases of animals pays a portion of the bacteriological laboratory expenses, and the Board is entitled to whatever work in that line it may need. We have two first-class bacteriologists and a number of assistants at work in a finely equipped laboratory.

As a rule, reports of infectious diseases of animals come first to the local health-officer of some town, or the chairman of township supervisors in the country, and our law provides that this local health officer shall report the same to the State Board of Health within twenty-four hours. Local authorities are expected to take immediate charge of outbreaks of infectious diseases under the direction of the veterinary department of the State Board. Local authorities employ their own veterinarians and pay their own bills, but the State Board of Health has rules which have been issued for their guidance, and the work for each outbreak is supervised by the director of the veterinary department, either by correspondence or by the assistance of a field-veterinarian.

We are quite proud of our new Minnesota law dealing with infectious diseases of domestic animals, and in order that I may make plain our methods of dealing with the hog-cholera problem it will be necessary to give some extracts from this law:

SECTION 1. Authority is hereby given to the State Board of Health and to the several local boards of health of the towns, villages, and cities of this State, to take all steps they may severally deem necessary to control, suppress, and eradicate any and all contagious and infectious diseases among any of the domestic animals in this State, and to that end, said boards are hereby severally empowered, within their respective jurisdictions, to quarantine any domestic animal which is infected with any such disease or which has been exposed to infection therefrom; to kill any animal so infected, and, whenever deemed necessary by the State Board of Health, to kill any animal which has been exposed to the infection of any such disease. Said State Board of Health is hereby expressly given authority to regulate or prohibit the shipment into this State of any domestic animal which, in the judgment of said board, may endanger the public health.

SEC. 2. Any person who knows of, or has reason to suspect, the existence of any contagious or infectious disease in any domestic animal, shall forthwith give notice thereof to the local board of health of the town, village, or city, where such animal is kept. Within twenty-four hours after any local board of health shall receive notice that any domestic animal is infected with any such disase, or has been exposed thereto, it shall give notice thereof in writing to the State Board of Health.

SEC. 4. No animal shall be killed by any of the boards herein mentioned until it shall first have been adjudged to be infected with a contagious or infectious disease, either by a duly authorized agent of the State Board of Health or by a physician or veterinary surgeon selected by a local board of health; except that, whenever, in the judgment of the State Board of Health, the control or eradication of a disease renders it advisable to do so, such board may order killed and buried, or otherwise destroyed, any domestic animal which has been exposed to a contagious or infectious disease, although at the time not infected therewith.

SEC. 7. It is hereby made the duty of the several local boards of health in this State to carry out and enforce all orders and directions of the State Board of Health to them directed, and the State Board of Health may require any two or more local boards to act together for the purpose of enforcing any of the provisions of this act.

SEC. 8. The State Board of Health, or any duly authorized agent thereof, may examine, or cause to be examined under oath, all persons believed to possess knowledge of material facts concerning the existence or dissemination, or danger of dissemination, of disease among domestic animals; and, for this purpose, shall have all the powers vested in justices of the peace to take depositions and to compel witnesses to attend and testify.

SEC. 9. Any person violating any provision of this act or any rule or regulation made by the State Board of Health, or by any local board of health, or any order made by any such board under the authority hereof, shall be guilty of a misdemeanor and be punished by a fine of not less than twenty-five or more than one hundred dollars, or by imprisonment for not less than thirty or more than ninety days. Any member of any local board of health who shall neglect or refuse to carry into effect the provisions of this act, or who shall neglect or refuse to carry out any directions of the State Board of Health, or who shall neglect or refuse to enforce any rule or regulation made by the State Board of Health, or by any local board of health, under the authority hereof, shall be guilty of a misdemeanor and be punished by a fine of not less than twenty-five and not more than one hundred dollars; and each and every day's neglect or refusal to perform any duty imposed upon him by this act shall constitute a separate and independent misdemeanor.

SEC. 11. Whenever during the prevalence in the State of any contagious or infectious disease among domestic animals the owner shall post on his premises a notice forbidding all persons not authorized by State or local boards of health to enter any building or enclosure on said premises without permission from said owner, it shall be a misdemeanor to enter upon said premises, punishable by a fine of not less than twenty-five dollars, nor more than one hundred dollars, or by imprisonment for not less than thirty nor more than ninety days.

### CHAPTER 47—H. F. NO. 358.

### AN ACT RELATING TO THE SPREAD OF DISEASE AMONG SWINE.

*Be it enacted by the Legislature of the State of Minnesota:*

SECTION 1. It shall be the duty of the owner, or of any other person having in charge any swine that have died of any disease, immediately upon the fact of such death by disease coming to his knowledge, to bury the same at least three feet below the surface of the ground, or burn the same so that the carcass is consumed. No person shall sell, give away, or order for sale any swine that have died of any disease or have been killed on account of any disease. No person shall convey upon or along any public highway, or other public ground, or any private land, except his own, any diseased swine, or swine that have died of or have been slaughtered on account of any disease. It shall be unlawful for any person negligently or wilfully to allow his hogs or those under his control afflicted with any disease to escape his control or run at large.

SEC. 2. Any person convicted of a violation of this act shall be fined in any sum not less than ten nor more than one hundred dollars, or by imprisonment in the county jail not to exceed thirty days.

SEC. 3. This act shall take effect and be in force from and after its passage.

Approved March 12, 1897.

I would call your attention to the fact that definite and full authority is given to local and State boards. This law makes it the duty of any person who knows or has reason to suspect the existence of any infectious disease among any domestic animals to report the same immediately to the local board, and provides penalties for disobedience. The State board has authority to order the killing and disposal of the carcass of any domestic animal which has been exposed to an infectious disease, although not at that time diseased. The State Board of Health or any duly authorized agent may examine or cause to be examined under oath any person believed to possess knowledge of material facts concerning any infectious disease among domestic animals. This law gives owners authority to post a notice according to Section 11, which notice amounts to private quarantine in self-defence against careless neighbors. The carcasses of swine that have died of any disease must be burned or buried as prescribed. It is unlawful to sell, offer for sale, or give away carcasses of swine that have died from any disease or have been killed on account of any disease. It is unlawful for any person to convey the carcasses of swine that have died of any disease, or been killed on account of any disease, along any public highway or any private land, except his own. It is unlawful for owners negligently or wilfully to permit hogs suffering from any disease to escape control and run at large. And, lastly, the fines that are imposed by this law are such that suit may be brought in a justice's court, and are so moderate that judges, justices, and juries will be willing to impose them.

### HOG-CHOLERA CAMPAIGN OF 1897.

Please bear in mind that this work did not begin until the summer of 1897, and at that time we probably had forty-one counties more or less infected with hog-cholera and a large mass of farmers and local health-officers to deal with who were intelligent but who knew very little of hog-cholera, and, as a rule, were indifferent to proposed methods of control.

Obviously the proper treatment for this case was to stir up and give the most information to the largest number of people in the shortest time. Considerable time and energy and a portion of our annual appropriation for 1897 were, therefore, devoted to this end. Local papers of the infected district were furnished a great deal of hog-cholera literature, and the editors kindly published it. About 500 papers in this district have been patiently helping us scatter information concerning hog-cholera. I sent return postal cards to township chairmen in nearly forty counties, partly for the purpose of getting needed information and partly for the purpose of stirring them up and getting them interested in the work. The newspapers have printed copies of our new report blanks, hog-cholera quarantine card, notice card, hog-cholera circular, etc. We distributed copies of the law and hog-cholera circular giving information concerning the disease freely throughout the infected territory. This circular deals with symptoms, causes of disease, how the disease is scattered, what to do when an outbreak occurs, mistakes made by farmers, etc.

Hog-cholera was discussed at a large number of farmers' institutes and other farmers' meetings throughout the southern portion of the State.

It has been our aim to get this work in such a shape that we might deal with hog-cholera as the sanitarian in the field of human medicine deals with smallpox—*i. e.*, by intelligent prevention or individual and carefully prescribed quarantine if the disease has appeared. In order fairly to estimate the results of this first campaign we must divide the State into two districts: (1) Previously and badly infected district, and (2) territory infected for the first time during 1897. In the former district, which included fifteen counties, our results were far from ideal, although the maps and records indicate that we have been able, among other things, to render the spread of disease less rapid. This is also true of the territory immediately bordering on the generally infected district. In territory infected for the first time during 1897 our results were much more satisfactory. In many cases the disease did not spread beyond the township in which it first appeared. Our records indicate that there were forty townships infected at some time between January 1st and November 1st, and in which the disease had disappeared before the latter date. Total number of counties infected during 1896, probably forty-one. Total number in which the disease appeared during 1897, thirty-nine.

That it is possible and practical to maintain successful individual farm quarantine against hog-cholera was demonstrated over and over again during our imperfect work of 1897. The two following letters illustrate this point:

*County of Hennepin, Township of Greenwood.*

Sanitary rules and regulations for the town were adopted on the 13th of April, to go into effect the 1st of May.

On or about the first day of December the State Board of Health called our attention to the fact of a communication to its office that hog-cholera existed in our town, and for us to quarantine. Upon investigation, it was found that an extremely fatal and apparently contagious disease had broken out among the hogs on twelve farms since last September, mostly in the northwestern part of the town, resulting in the loss of 241 hogs, and since quarantine, 37, making in all 278 hogs. At a conservative estimate, we deem that our people have lost between nine and ten hundred dollars by this disease. Quarantine was established by this board on the 4th of December, 1897, against the further spread of suspected hog-cholera, to continue in force until disinfection was made. In connection therewith an order was issued prohibiting the slaughter of hogs for either domestic use or transportation from the township, unless first inspected by some member of the town board. The number of hogs inspected by the members was 136.

At present, as far as we know, this contagion has ceased within the original bounds of quarantine (*no spread beyond quarantine limits*).

The expense incurred to the town for the control of hog-cholera will approximate twenty-two dollars.

We are of the candid opinion that if our people would promptly report to the Board of Health the first appearance of any dangerous contagion, before it gains an extensive foothod, it would be easier to check the ravages of such sickness, and would surely add to the general comfort and welfare of our townspeople. E. SIPE, C. B. S.

The following letter from Charles Kenning, Secretary of the Minnesota Swinebreeders' Association, illustrates again results that may be accomplished under reasonably favorable conditions by promptly quarantining outbreaks of hog-cholera:

OSCEOLA, March 1, 1898.

DEAR SIR: In reply to yours of the 18th inst., I would say that quarantine was established in Osceola Township, Renville County, on September 29th, at which time four farms were quarantined; but it was learned later that at least eight farms were infected at that time. This covered the eastern portion of the township, mostly settled by a class of people with whom it is difficult to deal in work of this kind. During the next two weeks the disease spread some in this neighborhood, as these farmers would visit back and forth and help one another without proper precaution. The remainder of the town insisted that these farmers in this infected district should keep at a safe distance, and they avoided the infected farms as much as possible. *There was not a case of hog-cholera outside the quarantined area*, and even in the infected district those that would not come in contact in any way with the infected farms also escaped. Although the local board did very well in this infected township, there were some things left undone. The loss would probably have been much less if the farmers, who insisted upon doing as they pleased, had been severely dealt with according to law. I am satisfied from our recent experience that much can be done by proper quarantine; but the people themselves must do their part, and if they refuse, all necessary force should be used to make them. In all, seventeen farms were quarantined in this township.

For the benefit of Iowa and Nebraska friends I would suggest that practically all of our trouble in this line came from these two States, and was usually distributed from some railroad stockyards in Minnesota.

In estimating our results for 1897 it should be considered that the methods then in use did not have a fair trial. The disease had extended over forty or forty-one counties when this work began. We had a lot of local health-officers to deal with who knew very little about hog-cholera and very little concerning their duties in connection with it.

### PRESENT METHODS—1898.

We have continued the general plan of individual farm-quarantine, and are trying to make it clear to farmers and stockmen that we quarantine only the hogs, hog-pens, yards, etc.—not the entire farm, as many of our people seemed to think last year. We are trying to get in as close touch as possible with the stockmen and railroad men of the State for very obvious reasons—we need their support and help.

Whenever any suspicious disease appears on any farm the township supervisors are expected to place all hogs, hog-pens, yards, etc., where hogs have been confined recently, under rigid quarantine. This quarantine must include all hogs that have been exposed directly or indirectly with those that are sick. Conditions of quarantine, specified on the hog-cholera card, read as follows:

All persons excepting the owner, duly authorized attendants, or medical advisers are forbidden to enter any inclosures where hogs are kept on these premises, until this card has been removed by permission from the State or local Board of Health.

Hogs must not be removed frcm these premises after date of this card, until six months after the last case has died or recovered, except in the following cases: 1st, by permission in writing given by the State Board of Health; and, 2d, dressed carcasses of healthy hogs killed under inspection of the State or local Board of Health.

No hogs, excepting those hereby quarantined and their offspring, shall be allowed upon these premises until six months after the last hog has died or recovered. During this period of six months no other domestic animal shall be permitted in these premises for any reason whatever.

Parties living on this place must not go near pens or yards where hogs are kept on other farms.

Keepers of these hogs will be held responsible for the unauthorized removal of this card and for allowing any swine hereby quarantined to escape from these pens or yards and run at large.

Township supervisors are also expected to warn neighboring farmers of the presence of the disease, and give them such other information as they may need. When the supervisors are in doubt as to the nature of the disease, it is their duty to call a competent veterinarian to make necessary examinations and to ascertain whether the disease is hog-cholera. The hog-cholera card is posted in conspicuous places about the pens and yards.

The general plan of an educational campaign has been continued, but in a somewhat different way. Printed matter is still distributed freely, and we are doing a lot of personal work with local health-officers. We now have two field-veterinarians, one of whom devotes his entire time to work with hog-cholera, and the other gives a portion of his time to it.

Dr. Annand's field work is probably somewhat unique. Early in the season he began a campaign of personal work among township supervisors, the plan being to visit the chairmen of boards of township supervisors, and talk hog-cholera with them; for instance, he takes dinner with one chairman, stays over night with the next one, and gives as much information as possible concerning the nature of the disease, how it usually spreads, duties of local health-officers, and gives specific instructions as to just what should be done in case hog-cholera appears in that township. He is doing this work in what we call the border counties—*i. e.*, just north of our generally infected district. Beginning in the eastern part of Wright County, he visited every township in a strip of country two townships wide across the southern portion of that county; all the townships in Meeker County, excepting the four northern ones, and a strip of territory four townships wide across the southern portion of Kandiyohi County. In a similar way the eastern, southern, and western portions of Stearns County were visited. The townships to be visited are selected according to location of infection last year; and in a similar way he will work through Chippewa, Swift, and Lac-qui-parle Counties. Dr. Annand is able to visit about two townships per day. He travels mainly by bicycle, and his expenses are reduced to a minimum, averaging thus far about eleven dollars per week, much less than we estimated before the work began. I believe this work is one of the best things so far attempted. I do not suppose that all the chairmen of town boards in this territory will do ideal work if the disease comes their way, but I do believe that they will know enough about to it take prompt action and at least report the first outbreak, so that they can be given specific instructions for further work. During our first season's work many of our local health-officers seemed to think an outbreak covering less than half a dozen farms was not worth while reporting.

Our placards, blanks, circulars, etc., are similar to those in use last year, but all have been modified more or less as experience has proved necessary. The "Notice" card has proven a good thing, and is continued. Farmers will probably use them much more freely this season than last, for they are beginning to realize the possibilities that lie in the way of private quarantine when supported by law.

Our stock-letters, as we call them, are printed with copying ink and in a color and style similar to the typewriter. These letters copy nicely in letter books. Each case usually calls for special comment or instruction, and space is left at the top for the address and this instruction in addition to the stock-letter, which fits all of these cases. We save an immense amount of office-work in this way. We have a letter of this kind for glanders, another for hog-cholera, and will soon have one for tuberculosis.

Our hog-cholera stock-letter has been modified and reads as follows:

It is the duty of local health-officers, including township supervisors, to quarantine all yards and pens in which there has recently been any suspicious swine-disease. Conditions of quarantine are given on the "Hog-cholera" and "Suspicious Swine-disease" card, sent you under separate cover.

I send you to-day a complete hog-cholera file, containing several copies of the "Blank for Reporting Infectious Diseases Among Domestic Animals." Please fill out one of these for each farm whereon the disease has appeared, and return to me as soon as possible. It is your duty to put up in a conspicuous place one or more of the "Hog-cholera" and "Suspicious Swine-disease" cards, bearing in mind that you are quarantining only the yards, sheds, pens, etc., where hogs have been confined. Please distribute copies of the law and hog-cholera circulars freely in this neighborhood. I think it would be wise for you to read the hog-cholera circulars and law carefully, that you may give the neighbors such advice as they need. Call their attention especially to the last section of the law and see that it is enforced.

The "Notice" cards should be distributed among neighbors whose hogs have not been sick, and who may wish to avail themselves of the protection which the law gives them: that is, the right to issue private quarantine in self-defence.

Neighbors should be warned of the presence of the disease and informed that it is very unwise for them to go where sick hogs have been kept on other farms; and equally dangerous to permit visiting neighbors to go into hog-pens or yards.

I hope your people will realize that hog-cholera is infectious, like small-pox or diphtheria, and must be conveyed from farm to farm, otherwise it does not spread.

The fact that there has been no sickness during the past few weeks among hogs on a farm where there has recently been an infectious swine-disease gives no assurance of safety. The farm may still be infectious, and should be so regarded. It may be necessary for you to watch this thing closely for several months. It is folly to waste valuable time in discussing the name of the disease that is now prevailing in your township. The name is the least important feature; but if it is infectious—*i. e.*, catching—it is hog-cholera, and must be treated as such.

As nearly as possible we send a field-veterinarian to each outbreak, with instructions to stay as long as necessary and to visit chairmen of all adjoining townships for the purpose of getting them ready for possible trouble.

The following regulations were adopted last summer and are now in force:

HOG-CHOLERA REGULATIONS.

All railroad shipping-pens in the following counties are hereby declared to be probable or possible sources of infection for hog-cholera: Fillmore, Moore, Freeborn, Faribault, Martin, Jackson, Nobles, Rock, Pipestone, Murray, Cottonwood, Watonwan, Blue Earth, Waseca, Steele, Dodge, Olmsted, Winona, Dakota, Scott, Sibley, Renville, Yellow Medicine, Lac-qui-parle, Chippewa, Kandiyohi, McLeod, Carver, Anoka, Stearns, Pope, and Swift.

1. Hogs must not be removed from any railroad shipping-pen located within the aforesaid counties, except for shipment by rail to some point for slaughter.

2. Hogs shipped from point to point in Minnesota, or from another State into Minnesota, and not intended for immediate slaughter or exhibition at the State fair, must be crated, shipped in other than stock-cars, and accompanied by a certificate stating that they were free from disease when shipped, and that there had been no hog-cholera in the neighborhood from which they were shipped for a period of at least six months previous to shipment. This certificate must be signed by a licensed physician, veterinarian or health-officer, and must be delivered to the local health-officer of the district into which the hogs are shipped.

3. Hogs for shipment in crates must not be permitted in or loaded from railroad shipping-pens.

4. Hogs intended for exhibition at the State fair must be shipped in cars that have never carried hogs or in stock-cars that have been disinfected by the railroad according to agreement with the State Board of Health. They must be shipped in crates, and must not be loaded from or through any railroad shipping-pens. Upon arrival at the fair-grounds and before unloading, the person in charge shall be required to sign a certificate stating that the hogs were free from disease when shipped and that there had been no hog-cholera in the neighborhood for a period of at least six months previous to shipment. Managers of county and district fairs held in any of the counties named above are requested not to have swine exhibits in connection with such fairs during 1898.

### SWINE EXHIBITS AT STATE AND COUNTY FAIRS.

These have furnished us a difficult problem. Some of the conditions imposed last year were plainly impracticable, and our plans for 1898 are considerably different. This fall we made such arrangements with general freight-agents that swine for exhibition at the State fair came in other than stock-cars, or in stock-cars that had never carried hogs, or in stock-cars that had been especially disinfected and provided for this purpose. Ten days before the fair opened general freight-agents were furnished with a complete list of all intending swine-exhibitors, and they then provided sufficient horse-cars or new stock-cars, or stock-cars that had been disinfected by steam in the shops for this special work. Each freight-train passing through places from which swine shipments were to be made carried two such cars on Thursday, Friday, and Saturday before the fair. Upon arrival at the fair-grounds each exhibitor was required to sign a certificate to the effect that his hogs were free from disease when shipped, and came from a neighborhood in which there had been no suspicious swine-disease during the past six months. All hogs were inspected on their arrival by a representative of the State Board of Health, and thereafter daily during the fair.

Pens have been so altered that visitors cannot climb into them, and an extra partition, made tight, has been placed between pens, so there can be no possibility of manure or litter, or other possible infectious material, passing through one pen to the other.

I have been furnished with a list of exhibitors, and all swine exhibits will be followed to their homes by correspondence, that we may know whether our work succeeded. A small circular has been issued to exhibitors, and through the general offices to the local railroad agents, informing them of the conditions that have been imposed. This circular also informs exhibitors of the precautions that have been taken for their interests. Our rules provide further, that if daily inspection discovered any sick hogs, such hogs were to be isolated in pens especially provided. If the disease proved to be hog-cholera all sick hogs were to be promptly killed and the carcasses burned.

At the larger stockyards, for instance, South St. Paul and New Brighton, where government inspection is maintained, we coöperate with the Federal Government by giving government inspectors authority as representatives of the State Board of Health. This plan has proved satisfactory with several outbreaks of sheep-scab and hog-cholera which appeared in these yards. The following blank, when filled out by such representatives of the bureau and State Board of Health, gives needed information as to the place from which such diseased stock was shipped, township in which they were fed, etc. In this way it is possible to trace diseased animals back to the township from which they came:

Infectious Diseases of Animals. MINNESOTA STATE BOARD OF HEALTH.
F. VIII. 3-7-98 1M.

REPORT OF DISEASED ANIMALS RECEIVED AT STOCKYARDS.

Disease . . .
Point of shipment . . .
Number and kind of animals . . .
Fed by . . .
Fed in township of . . .
Consignor . . .
Consignee . . .
Number of diseased animals . . .
Disposition of diseased animals . . .
Remarks . . .
Signed, . . . Inspector.

NOTE.—Please report promptly in duplicate to the Veterinary Department of the Minnesota State Board of Health.

I might suggest for the benefit of others who are trying to solve some of these problems in State medicine, that we work in close coöperation with the city health departments of the larger cities. This plan has proved very economical and satisfactory. One inspector in Minneapolis and another in St. Paul are given authority as agents of the State Board of Health, so that in case diseased animals are taken out of quarantine or removed into the country before quarantine can be established the city representatives can follow them without a lot of red-tape correspondence with the State Board.

The hog-cholera situation in Minnesota at this date (August 15, 1898) is much more encouraging than at this time last year. Comparatively few outbreaks have appeared at this date, although I take it for granted that we will have trouble during September and October, when stock-buyers and threshing-crews are abroad and farmers have commenced fall-feeding. I have no means of knowing just what relation exists between hog-cholera and conditions of food and care, but I have a lingering suspicion that when the hog-cholera chapter is finally written there will be some things other than bacilli in it.

I take pleasure in showing you two maps, one for 1897 and the other for 1898. These maps show by the sizes and colors of the painted spots the location and period during which outbreaks occurred. I am aware that hog-cholera is less prevalent in other States than it was in 1897, and yet I think it is probable that our plan of interesting and educating farmers, and especially local health-officers, along hog-cholera lines, in addition to quarantine, has contributed materially to our present condition. The number of counties does not represent the number of outbreaks, for one outbreak may involve corners of four different counties, or an outbreak in the eastern side of one county may extend into the western side of another. During July and the first part of August, 1897, the reports came in rapidly, and we were very busy.

Perhaps some one is already saying, "But do you really consider individual-farm quarantine for hog-cholera a practical thing in extensive outbreaks?" I do consider such individual-farm quarantine entirely practical in recent and limited outbreaks. It can be made successful in extensive outbreaks, providing we have chairmen of townships who are active and willing to do considerable work for comparatively small pay, and do their duty in spite of opposition, and provided further that these boards of supervisors have the support of their influential and intelligent farmers.

We have been able to find such conditions in Minnesota quite frequently, but there have been exceptions. I do not suppose for a moment that this work is ideal, and yet we have accomplished results that have been fairly satisfactory to ourselves and to the stock-interests of the State. When farmers are generally convinced that hog-cholera is an infectious disease, and are better informed concerning it, and when township supervisors are better informed concerning their duties, our work will be easier and more satisfactory.

It is quite reasonable to suppose that in the near future we will be provided with a vaccine that can be economically produced, concentrated, conveniently administered, and thoroughly practical. When this happy day arrives we will have means that can be used to great advantage in connection with such quarantine as has been outlined in this paper. If an outbreak appears on a certain farm, all hogs on neighboring farms for several miles can be promptly vaccinated. In this way we will not only have all the safeguards that may come from quarantine, but we will also be able to "backfire," as it were, against the disease.

